# Effect of Electrode Shape on Impedance of Single HeLa Cell: A COMSOL Simulation

**DOI:** 10.1155/2015/871603

**Published:** 2015-04-16

**Authors:** Min-Haw Wang, Wen-Hao Chang

**Affiliations:** Department of Electrical Engineering, Chinese Culture University, Taiwan

## Abstract

In disease prophylaxis, single cell inspection provides more detailed data compared to conventional examinations. At the individual cell level, the electrical properties of the cell are helpful for understanding the effects of cellular behavior. The electric field distribution affects the results of single cell impedance measurements whereas the electrode geometry affects the electric field distributions. Therefore, this study obtained numerical solutions by using the COMSOL multiphysics package to perform FEM simulations of the effects of electrode geometry on microfluidic devices. An equivalent circuit model incorporating the PBS solution, a pair of electrodes, and a cell is used to obtain the impedance of a single HeLa cell. Simulations indicated that the circle and parallel electrodes provide higher electric field strength compared to cross and standard electrodes at the same operating voltage. Additionally, increasing the operating voltage reduces the impedance magnitude of a single HeLa cell in all electrode shapes. Decreasing impedance magnitude of the single HeLa cell increases measurement sensitivity, but higher operational voltage will damage single HeLa cell.

## 1. Introduction

As the severity of diseases increases, many researchers have begun investigating ways to reduce the death rate by curing diseases in early stages. In the case of diseases such as cancer, the conventional cell inspection rarely provides sufficient information for diagnosis because only a small percentage of cells exhibit symptoms of malfunction in early stages of these diseases [1, 2]. The conventional inspection uses average values for cellular parameters and cannot exactly represent individual cells [3–5]. Additionally, multiple parameters must be measured in single living cells to correlate cellular events and thus understand complex cellular processes [5, 6]. Hence, single cell analysis is an important trend in biological and medical research.

For single cell analysis, cell impedance analysis [7–10] has rapidly developed as an effective method of biological measurement. Impedance measurements can provide accurate and detailed information about electrical characterizations on single cells than those on pathological tissues. In the case of diseases such as cancer, biochemical functions of living biological cells change appear earlier than other clinical symptoms. Detecting these changes in the incubation stage is favorable to the general survey, prevention, and early stage treatment of the diseases [1, 2]. When used to monitor the change of biochemical functions of living biological cells in the period of the treatment and rehabilitation of diseases, cell impedance analysis provides a functional model that can be used to evaluate cells by applying physiology and pathology information [11]. Understanding single cell impedance variation could be helpful to realize the status of single cell. Hence, single cell impedance analysis is an effective method for evaluating treatment results or the stage of recovery [12]. Moreover, cell impedance analysis is applicable for studying effects of pharmaceutical compounds, viral and bacterial infections, environmental parameters, toxicity, and other factors on cells.

Most studies of single cell impedance have focused on impedance measurement and analysis. Ayliffe et al. used microchannels with integrated gold electrodes to measure electric impedance in air, in biological cells, and in various concentrations of PBS [13]. Cho et al. presented a novel biosensor for electrical/physical characterization of single cells [1]. The authors identified significantly different magnitudes and phase shifts between normal and abnormal red blood cells. Cho and Thielecke used a micro-hole-based cell chip to measure impedance in single L929 cells in various physical/chemical environments [14]. Jang and Wang used a microfluidic device to capture single cells and to measure impedance at various operational voltages and frequency [15]. However, all of the above studies encountered measurement problems that reduced the accuracy of their measurement results. Some problems were caused by the geometry of the detection electrodes in microfluidic devices. Electrodes have an important role in cell impedance measurements in microfluidic devices. Iliescu et al. used microfluidic devices with three different electrode geometries to measure phosphate buffer saline (PBS), living cells, and dead cells [16]. The electric field distribution affects the impedance measurement, and the electrode geometry affects the electric field distributions [17]. Therefore, optimizing the electrode shape can improve analysis of single cell behavior.

For a clear and in-depth understanding of the measured data and the biophysics behind the experimental phenomenon, analyzing the effects of electrode geometry on microfluidic devices is essential. This study performed numerical simulations of single cell impedance in electrodes with four different shapes. An equivalent electrical circuit model is used to obtain the numerical results from the FEM simulation of the COMSOL multiphysics package [18]. The detailed analytical results for impedance and phase of single HeLa (human cervical epithelioid carcinoma) cells at voltages ranging from 0.1 to 1.0 V and frequencies ranging from 5 to 100 kHz are also presented.

## 2. Theory

**Figure 1:**
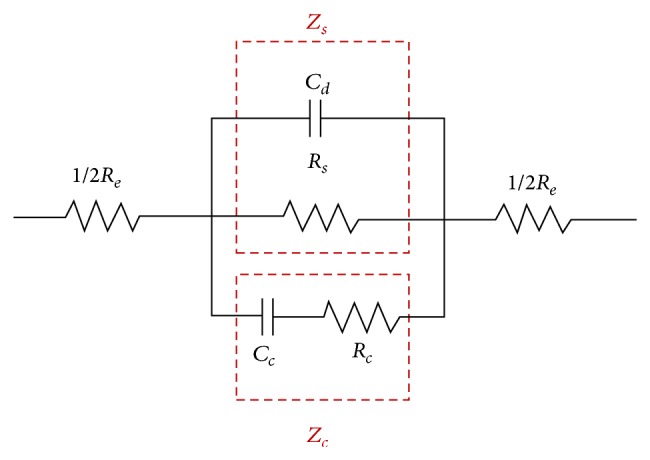
The equivalent circuit model of the system.

An authoritative electrical-biological-circuit-system is used in this study [18]. The system includes cell impedance *Z*_*c*_, PBS solution impedance *Z*_*s*_, and a pair of electrodes resistor *R*_*e*_ as shown in Figure 1. The *Z*_*c*_ is a capacitance of cell membrane *C*_*c*_ in series with a resistor cytoplasm *R*_*c*_. The *Z*_*s*_ represents the impedance contributed by all materials between the two electrodes, including the solution resistor *R*_*s*_ in parallel with capacitance of double layer *C*_*d*_. The circuit model is *Z*_*c*_ in parallel with *Z*_*s*_ and both are in series with *R*_*e*_. Additionally, this model is operated at frequency range between 5 and 100 kHz and voltage range from 0.1 V to 1 V. The overall impedance of the system can be written as

(1) Z=Re+11/Rs+jωCd+1/Rc+1/jωCc=ZReal+jZImg,

where *Z*_Real_ and *Z*_Img_ are real and imaginary parts of *Z*, respectively. The magnitude and angle of *Z* are given by (2) and (3), respectively:

(2) $$\begin{array}{c} Mag=\sqrt{Z_{\text{Real}}^{2}+Z_{\text{Img}}^{2}}, \end{array}$$

(3) $$\begin{array}{c} \theta =\tan^{-1}\frac{Z_{\text{Img}}}{Z_{\text{Real}}}. \end{array}$$

## 3. FEM Simulation

**Figure 2:**
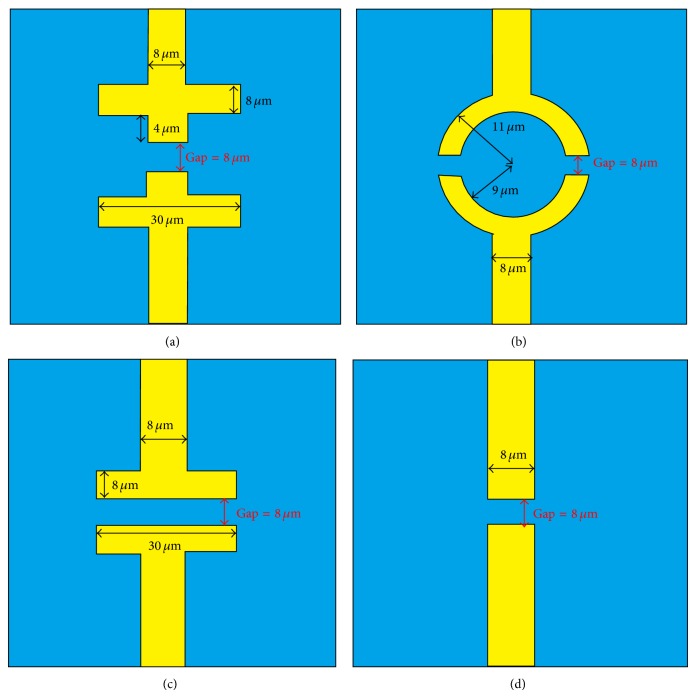
The four electrode shapes: (a) cross, (b) circle, (c) parallel, and (d) standard.

The COMSOL multiphysics commercial software package is used for electrical characterization of the biosensor system in this study. The program simulates electrical components (conductivity and permittivity) and devices used in electrostatic, magnetic static, and electromagnetic quasistatic applications, particularly in terms of the effects of other physical properties. The two-dimensional numerical model used in this study incorporates an AC/DC module. The width and length of the COMSOL structure are 100 *μ*m and 100 *μ*m, respectively. The origin of coordinate is in the center of COMSOL structure. In order to improve the sensitivity and electric field distribution of impedance measurement, four different electrode shapes have been proposed in this study.  Figure 2 shows the four different electrode shapes (cross, circle, parallel, and standard). The electrode gap is 8 *μ*m and the electrode is composed of gold (Au). The conductivity and relative permittivity of phosphate buffer saline (PBS) are 2 × 10^−6^ S/m and 136, respectively [15]. Tables 1 and 2 list the conductivity and relative permittivity of single HeLa cell at frequencies form 5 to 100 kHz and at voltages from 0.1 V to 1 V [18].

**Figure 3:**
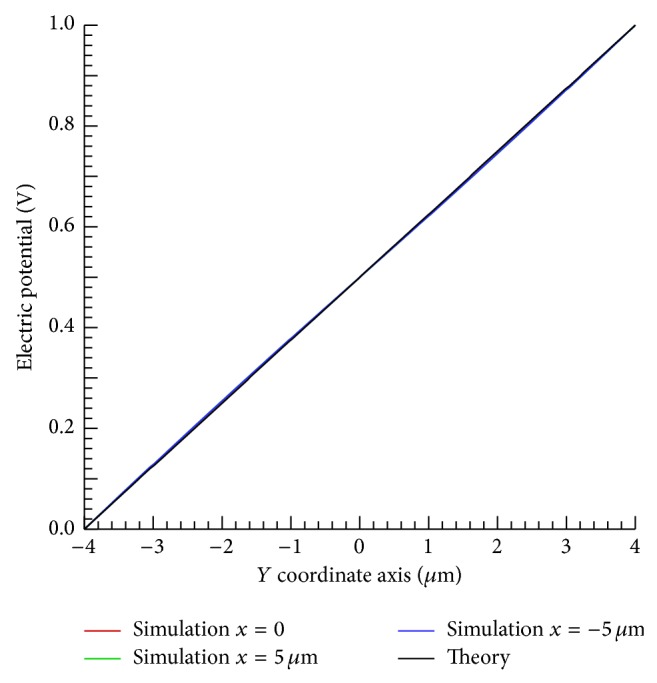
Variation of electric potential without the cell between electrodes in *y*-axis direction.

The accuracy of the simulation results was confirmed by comparing the electric potential of theory and simulation using parallel electrode.  Figure 3 plots the electric potential of theory and simulation result between electrodes. The simulation results are in almost perfect agreement with the theoretical results. The electric potential of simulation only changes on the *y* coordinate axis, which is also consistent with electric potential theory.

## 4. Results

This work used the COMSOL software package for electrical characterizations of single HeLa cells with four different electrode shapes. In the simulations, the frequency range was 5 to 100 k Hz and the voltage range was 0.1 to 1 V. The assumptions in the simulations were a single HeLa cell with a diameter of 20 *μ*m, which approximates the actual size [19, 20].

### 4.1. Electric Field Distributions

**Figure 4:**
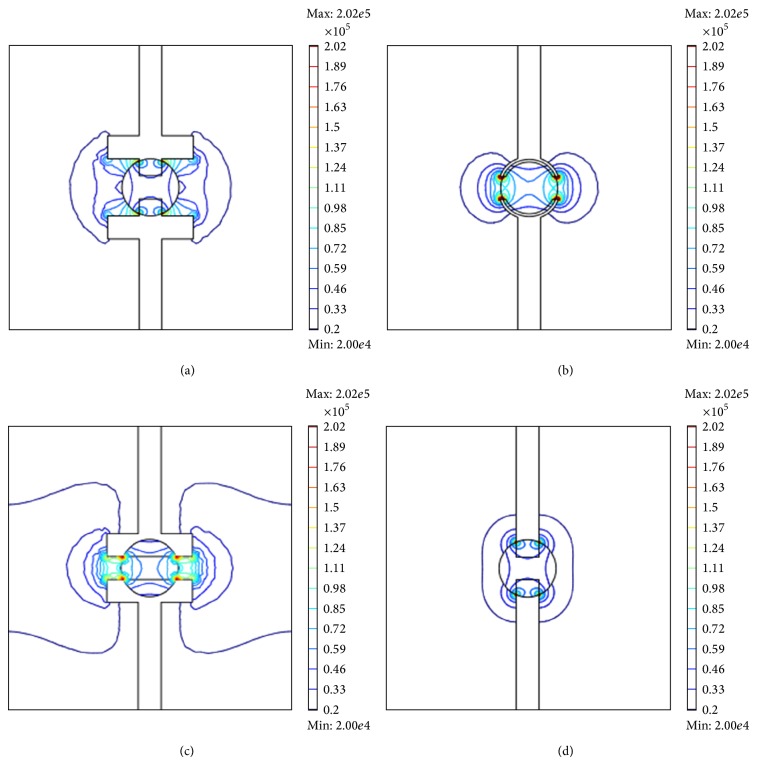
Schematic of four different electrodes and the electric field distributions induced in the vicinity of the electrode pair for a frequency of 100 kHz and an operating voltage of 1 V. (a) Cross electrodes, (b) circle electrodes, (c) parallel electrodes, and (d) standard electrodes.

Accurate single cell measurements require a uniformity electric field distribution in microfluidic devices [21]. Single cell impedance measurements can be affected by a highly varying electric field [22].  Figure 4 presents the distribution of electric field for the four different electrode shapes at operational voltage of 1 V and frequency of 100 kHz. The single HeLa cell has a radius of 10 *μ*m and is located in the center of the structure. Regardless of the electrode shape, the electric field strength is the highest near the region of boundary between cell and electrodes. The electric field strength of the circle and parallel electrodes are higher than those of the cross and standard electrodes. In the case of the circle and parallel electrode, the extracellular variation in the electric field intensity in the *x*-axis is higher than that of the intracellular variation. However, in the case of the cross and standard electrodes, intracellular variation in the electric field intensity in the *x*-axis direction is higher than that of the extracellular variation. The electrode shape caused the opposite behavior. In the case of the cross and standard electrodes, the electrodes are near the cell center.

**Figure 5:**
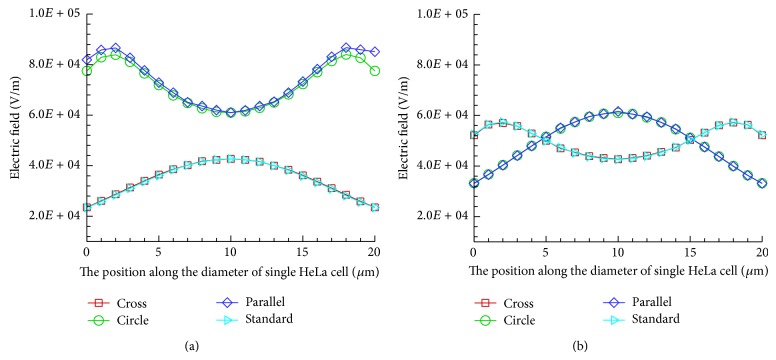
Distributions of electric field along the cell diameter. (a) *x*-axis and (b) *y*-axis.

Figure 5 presents the electric field distributions with the cell in the *x*-axis and *y*-axis for the four different electrode shapes at an operational voltage of 1 V and a frequency of 100 kHz. The simulation results for the electric field in the *x*-axis direction indicated that, of the four electrode shapes, the intensity of the electric field is the highest in the parallel electrodes. The distribution of electric field with cross electrodes resembles that of the standard electrodes, and the distribution of electric field with circle electrodes resembles that of the parallel electrodes. The similar contact region of the electrode and the single HeLa cells causes a similar electric field distribution. The electric field intensity is higher in the cell center than at the cell edge in both the cross and standard electrodes, but the electric field intensity is lower in the center than at the edge of the cell in the circle and parallel electrodes. The contact region of the cell and electrode determines the electric field distribution within the cell. The intensity of the intracellular electric field in the *x*-axis direction increases from 2.4 × 10^4^ to 4.3 × 10^4^ V/m for cross electrodes and that of the extracellular electric field in the *x*-axis direction decreases from 8.4 × 10^4^ to 6.1 × 10^4^ V/m for circle electrodes. The electric filed intensity of circle electrodes is higher than that of cross electrodes around 2 times in the intracellular at an operational voltage of 1 V and a frequency of 100 kHz. Additionally, the uniformity of the electric field distribution for the four different electrode shapes is similar in *y*-axis direction.

### 4.2. Impedance Variation

**Figure 6:**
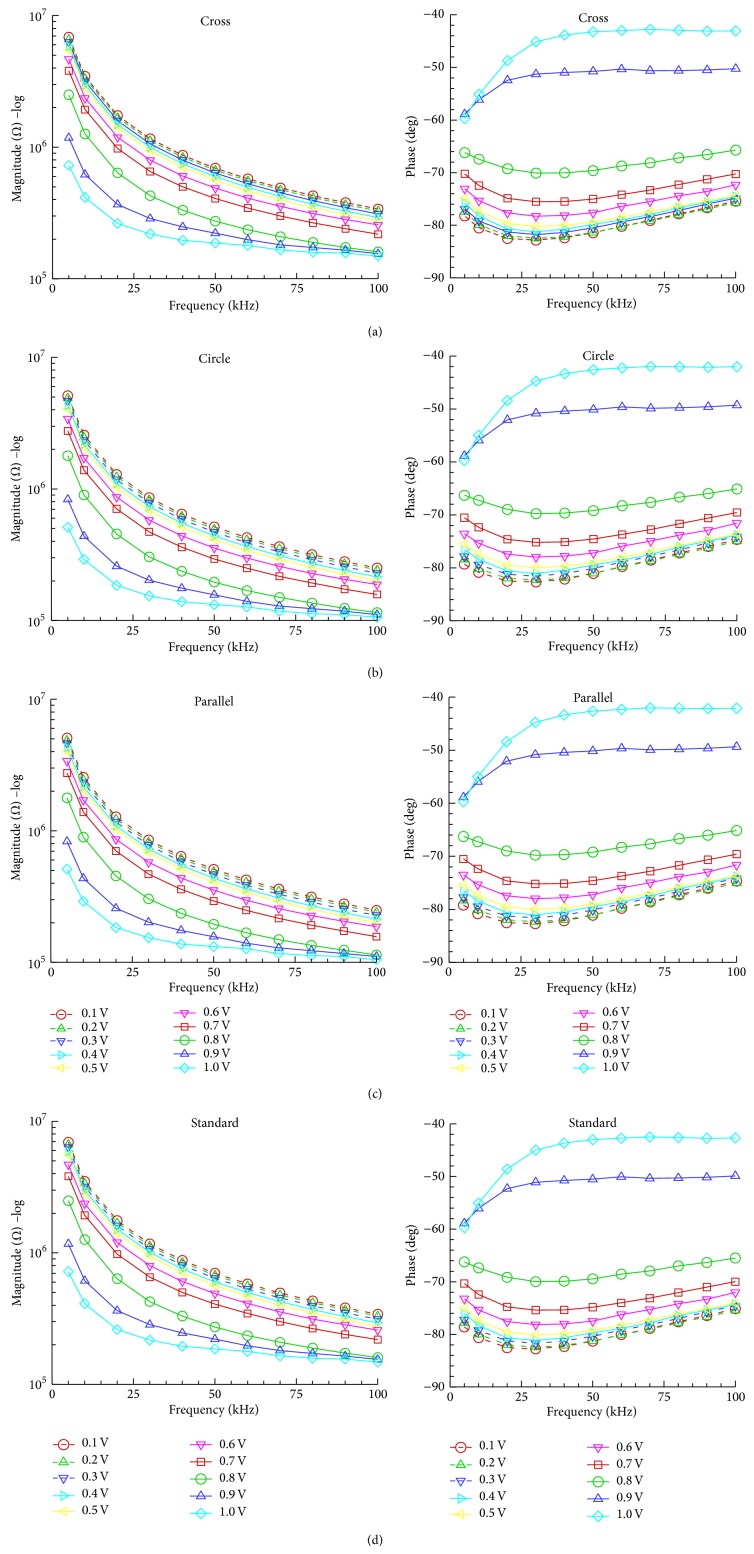
Impedance magnitude and phase of single HeLa cell at operating voltages ranging from 0.1 to 1.0 V. (a) Cross electrodes, (b) circle electrodes, (c) parallel electrodes, and (d) standard electrodes.

The equivalent circuit model of the system, including *Z*_*c*_, *Z*_*s*_, and *R*_*e*_, as shown in Figure 1, is used to obtain the magnitude and phase of impedance.  Figure 6 presents the simulation results for magnitude and phase of single HeLa cell impedance for the four different electrode shapes at operating voltage range from 0.1 to 1 V and frequency range from 5 to 100 kHz. Notably, the impedance results for operational frequencies lower than 5 kHz are omitted in the figures since the double layer may prevent the prediction of the cell conductivity at such low frequencies [18]. As observed in the electric field simulation, the cross and standard electrodes have similar impedance, and the circle and parallel electrodes have similar impedance. Regardless of electrode shape, an increased frequency reduces the magnitude of single HeLa cell impedance at all operational voltages because single HeLa cell was capacitive. The circle electrode simulations indicate that the magnitude of the single HeLa cell impedance decreases. The circle electrode simulations indicate that the single HeLa cell impedance decreases from 2.6 × 10^7^ to 2.5 × 10^5^ at frequency range from 1 to 100 kHz and operating voltage of 0.1 V. Additionally, an increased operating voltage reduces the single HeLa cell impedance in all electrode shapes. The circle electrode simulation results indicate that the magnitude of the single HeLa impedance decreases from 2.3 × 10^6^ to 1.1 × 10^5^ when the voltage is between 0.1 and 1 V and when the frequency is 100 kHz. A strong electric field may open the ionic channels of the cell membrane, which increases the ion exchange between the cytoplasm and the isotonic solution [23]. Therefore, the electrical resistivity of the cells declines, and the dielectric constant of the cells increases. The simulation results for the electrode shapes indicate that the magnitude value of the single HeLa cell is smaller for the circle and parallel electrodes than for the cross and standard electrodes because the electric field strength of circle and parallel electrodes is higher than that of cross and standard electrodes. Additionally, none of the four electrode shapes showed a large change in phase.

### 4.3. Sensitivity

**Figure 7:**
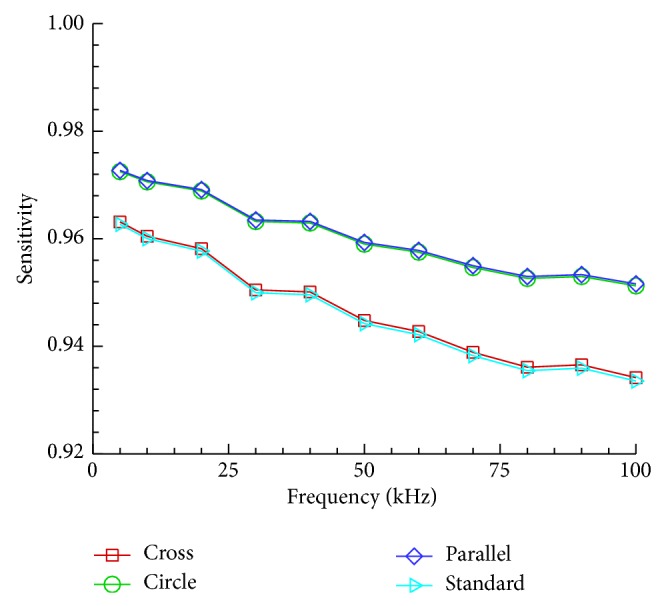
Comparison of sensitivity in the four electrode shapes at an operating voltage of 0.1 V and frequency range from 1 to 100 kHz.

Impedance analysis is an effective method of characterizing single cells based on their electrical response. Sun et al. presented the following alternative method for calculating impedance sensitivity [24]:

(4) $$\begin{array}{c} \text{Sensitivity}=\frac{Z_{\text{Solution}}-Z_{\text{single}\;\text{HeLa}\;\text{cell}}}{Z_{\text{Solution}}}, \end{array}$$

where *Z*_Solution_ is the impedance magnitude of the detection volume containing PBS and *Z*_single  HeLa  cell_ is the impedance magnitude of the HeLa cell and PBS in the detection volume.  Figure 7 shows the sensitivities for the four different electrode shapes at an operating voltage of 0.1 V and frequency range from 5 to 100 kHz. The circle and parallel electrodes have better sensitivity compared to the cross and standard electrodes. The sensitivity of the parallel electrodes decreases from 0.97 to 0.95 at a frequency range from 1 to 100 kHz and at an operating voltage of 0.1 V. As the strength of the electric field increases, the impedance of the single HeLa cell decreases. Therefore, circle and parallel electrodes have better sensitivity. However, it must not be forgotten that the magnitude of the useful signal also depends on the electric field intensity. Hence, increasing the sensitivity of measurement system could also reduce the signal-to-noise ratio and thus worsen the overall performance [16].

## 5. Conclusions

Numerical solutions were obtained by FEM simulations in the COMSOL multiphysics package to analyze the effects of electrode geometry on microfluidic devices into the impedance properties of single HeLa cell. An equivalent circuit model incorporating the PBS solution, a single HeLa cell, and a pair of electrodes is used to obtain the impedance of a single HeLa cell. The circuit model is *Z*_*c*_ in parallel with *Z*_*s*_ and both are in series with *R*_*e*_. The equivalent circuit model was used in COMSOL simulations to investigate how the magnitude and phase of a single HeLa cell are affected by electrodes with varying geometries, varying operational voltages, and varying frequency. The numerical solutions obtained by the COMSOL simulations indicate that the electric field strength of circle and parallel electrodes are higher than those of the cross and standard electrodes because the single HeLa cell magnitude value is smaller in the circle and parallel electrodes compared to the cross and standard electrodes. Simulations of different electrode shapes indicate that the magnitude of single HeLa cell impedance falls between 0.1 and 1 V because of decreasing electric field strength. The magnitude of single HeLa impedance decreases from 2.3 × 10^6^ to 1.1 × 10^5^ when the voltage is 0.1 to 1 V and the frequency is 100 kHz. Additionally, increasing the frequency reduced the impedance of the single HeLa cell in all electrodes shapes and at all operating voltages because the single HeLa cell was capacitive. At the same operating voltage, the circle and parallel electrodes provide higher electric field strength compared to the cross and standard electrodes. However, the cross and standard electrodes had a more uniform electric field distribution in the measurement environment. The circle and parallel electrodes also have better sensitivity compared to the cross and standard electrodes. Stronger electric fields cause greater impedance magnitude of single HeLa cell drop. Therefore, the circle and parallel electrodes have better sensitivity.

## Figures and Tables

**Table 1:** Conductivity of single HeLa cell at frequency range between 5 and 100 kHz and voltage range from 0.1 V to 1 V.

Frequency (kHz)	*σ* (0.1 V)	*σ* (0.2 V)	*σ* (0.3 V)	*σ* (0.4 V)	*σ* (0.5 V)	*σ* (0.6 V)	*σ* (0.7 V)	*σ* (0.8 V)	*σ* (0.9 V)	*σ* (1.0 V)
5	2.25 × 10^−4^	2.60 × 10^−4^	2.95 × 10^−4^	3.60 × 10^−4^	4.25 × 10^−4^	5.90 × 10^−4^	8.85 × 10^−4^	1.70 × 10^−3^	4.80 × 10^−3^	7.64 × 10^−3^
10	4.25 × 10^−4^	4.80 × 10^−4^	5.55 × 10^−4^	6.60 × 10^−4^	7.75 × 10^−4^	1.09 × 10^−3^	1.64 × 10^−3^	3.30 × 10^−3^	9.95 × 10^−3^	1.53 × 10^−2^
20	7.30 × 10^−4^	8.20 × 10^−4^	9.55 × 10^−4^	1.10 × 10^−3^	1.32 × 10^−3^	1.90 × 10^−3^	2.88 × 10^−3^	6.10 × 10^−3^	1.85 × 10^−2^	2.80 × 10^−2^
30	1.10 × 10^−3^	1.20 × 10^−3^	1.40 × 10^−3^	1.60 × 10^−3^	1.92 × 10^−3^	2.75 × 10^−3^	4.18 × 10^−3^	8.80 × 10^−3^	2.43 × 10^−2^	3.60 × 10^−2^
40	1.60 × 10^−3^	1.70 × 10^−3^	2.00 × 10^−3^	2.30 × 10^−3^	2.65 × 10^−3^	3.70 × 10^−3^	5.50 × 10^−3^	1.14 × 10^−2^	2.83 × 10^−2^	4.10 × 10^−2^
50	2.30 × 10^−3^	2.40 × 10^−3^	2.80 × 10^−3^	3.20 × 10^−3^	3.55 × 10^−3^	4.80 × 10^−3^	7.00 × 10^−3^	1.41 × 10^−2^	3.18 × 10^−2^	4.35 × 10^−2^
60	3.20 × 10^−3^	3.30 × 10^−3^	3.80 × 10^−3^	4.20 × 10^−3^	4.70 × 10^−3^	6.30 × 10^−3^	8.73 × 10^−3^	1.71 × 10^−2^	3.60 × 10^−2^	4.55 × 10^−2^
70	4.20 × 10^−3^	4.30 × 10^−3^	4.90 × 10^−3^	5.40 × 10^−3^	6.00 × 10^−3^	7.80 × 10^−3^	1.06*E* − 02	1.98 × 10^−2^	3.90 × 10^−2^	4.93 × 10^−2^
80	5.40 × 10^−3^	5.50 × 10^−3^	6.20 × 10^−3^	6.90 × 10^−3^	7.50 × 10^−3^	9.50 × 10^−3^	1.27 × 10^−2^	2.28 × 10^−2^	4.10 × 10^−2^	5.13 × 10^−2^
90	6.70 × 10^−3^	6.80 × 10^−3^	7.70 × 10^−3^	8.40 × 10^−3^	9.10 × 10^−3^	1.11 × 10^−2^	1.49 × 10^−2^	2.56 × 10^−2^	4.30 × 10^−2^	5.20 × 10^−2^
100	8.20 × 10^−3^	8.30 × 10^−3^	9.30 × 10^−3^	1.01 × 10^−2^	1.09 × 10^−2^	1.31 × 10^−2^	1.72 × 10^−2^	2.86 × 10^−2^	4.59 × 10^−2^	5.49 × 10^−2^

**Table 2:** Relative permittivity of single HeLa cell at frequency range between 5 and 100 kHz and voltage range from 0.1 V to 1 V.

Frequency (kHz)	*ε* (0.1 V)	*ε* (0.2 V)	*ε* (0.3 V)	*ε* (0.4 V)	*ε* (0.5 V)	*ε* (0.6 V)	*ε* (0.7 V)	*ε* (0.8 V)	*ε* (0.9 V)	*ε* (1.0 V)
5	5.00 × 10^3^	5.20 × 10^3^	5.50 × 10^3^	5.90 × 10^3^	6.30 × 10^3^	7.50 × 10^3^	9.20 × 10^3^	1.40 × 10^4^	2.85 × 10^4^	4.70 × 10^4^
10	5.00 × 10^3^	5.20 × 10^3^	5.50 × 10^3^	5.90 × 10^3^	6.30 × 10^3^	7.50 × 10^3^	9.20 × 10^3^	1.40 × 10^4^	2.62 × 10^4^	3.90 × 10^4^
20	5.00 × 10^3^	5.20 × 10^3^	5.50 × 10^3^	5.90 × 10^3^	6.30 × 10^3^	7.50 × 10^3^	9.20 × 10^3^	1.40 × 10^4^	2.10 × 10^4^	2.80 × 10^4^
30	5.00 × 10^3^	5.20 × 10^3^	5.50 × 10^3^	5.90 × 10^3^	6.30 × 10^3^	7.50 × 10^3^	9.20 × 10^3^	1.40 × 10^4^	1.75 × 10^4^	2.10 × 10^4^
40	5.00 × 10^3^	5.20 × 10^3^	5.50 × 10^3^	5.90 × 10^3^	6.30 × 10^3^	7.40 × 10^3^	9.20 × 10^3^	1.35 × 10^4^	1.50 × 10^4^	1.70 × 10^4^
50	5.00 × 10^3^	5.20 × 10^3^	5.50 × 10^3^	5.90 × 10^3^	6.30 × 10^3^	7.30 × 10^3^	8.80 × 10^3^	1.30 × 10^4^	1.33 × 10^4^	1.40 × 10^4^
60	5.00 × 10^3^	5.20 × 10^3^	5.50 × 10^3^	5.90 × 10^3^	6.30 × 10^3^	7.20 × 10^3^	8.60 × 10^3^	1.25 × 10^4^	1.23 × 10^4^	1.20 × 10^4^
70	5.00 × 10^3^	5.20 × 10^3^	5.50 × 10^3^	5.90 × 10^3^	6.30 × 10^3^	7.10 × 10^3^	8.43 × 10^3^	1.20 × 10^4^	1.15 × 10^4^	1.10 × 10^4^
80	5.00 × 10^3^	5.20 × 10^3^	5.50 × 10^3^	5.90 × 10^3^	6.30 × 10^3^	7.00 × 10^3^	8.26 × 10^3^	1.15 × 10^4^	1.05 × 10^4^	1.00 × 10^4^
90	5.00 × 10^3^	5.20 × 10^3^	5.50 × 10^3^	5.90 × 10^3^	6.30 × 10^3^	6.85 × 10^3^	8.09 × 10^3^	1.11 × 10^4^	9.70 × 10^3^	9.00 × 10^3^
100	5.00 × 10^3^	5.20 × 10^3^	5.50 × 10^3^	5.90 × 10^3^	6.30 × 10^3^	6.70 × 10^3^	7.92 × 10^3^	1.07 × 10^4^	9.20 × 10^3^	8.50 × 10^3^
